# Progress in Testing for and Treatment of Hepatitis C Virus Infection Among Persons Who Inject Drugs — Georgia, 2018

**DOI:** 10.15585/mmwr.mm6829a2

**Published:** 2019-07-26

**Authors:** Ketevan Stvilia, Philip R. Spradling, Alexander Asatiani, Maka Gogia, Khatuna Kutateladze, Maia Butsashvili, Jaba Zarkua, Tengiz Tsertsvadze, Lali Sharvadze, Maia Japaridze, Tinatin Kuchuloria, Lia Gvinjilia, Irinka Tskhomelidze, Amiran Gamkrelidze, Irma Khonelidze, David Sergeenko, Shaun Shadaker, Francisco Averhoff, Muazzam Nasrullah

**Affiliations:** ^1^National Center for Disease Control and Public Health of Georgia, Tbilisi, Georgia; ^2^Division of Viral Hepatitis, National Center for HIV/AIDS, Viral Hepatitis, STD, and TB Prevention, CDC; ^3^Georgian Harm Reduction Network, Tbilisi, Georgia; ^4^Health Research Union, Tbilisi, Georgia; ^5^Hepatology and Gastroenterology Department, Medical Center Mrcheveli, Tbilisi, Georgia; ^6^Infectious Diseases, AIDS and Clinical Immunology Research Center, Tbilisi, Georgia; ^7^Hepatology Clinic HEPA, Tbilisi, Georgia; ^8^Foundation for Innovative New Diagnostics, Tbilisi, Georgia; ^9^Training Programs in Epidemiology and Public Health Interventions Network, Tbilisi, Georgia; ^10^Georgia Ministry of Health, Labour and Social Affairs, Tbilisi, Georgia.

In April 2015, the country of Georgia, with a high prevalence of hepatitis C virus (HCV) infection (5.4% of the adult population, approximately 150,000 persons), embarked on the world’s first national elimination program (*1*,*2*). Nearly 40% of these infections are attributed to injection drug use, and an estimated 2% of the adult population currently inject drugs, among the highest prevalence of injection drug use in the world (*3*,*4*). Since 2006, needle and syringe programs (NSPs) have been offering HCV antibody testing to persons who inject drugs and, since 2015, referring clients with positive test results to the national treatment program. This report summarizes the results of these efforts. Following implementation of the elimination program, the number of HCV antibody tests conducted at NSPs increased from an average of 3,638 per year during 2006–2014 to an average of 21,551 during 2015–2018. In 2017, to enable tracking of clinical outcomes among persons who inject drugs, NSPs began encouraging clients to voluntarily provide their national identification number (NIN), which all citizens must use to access health care treatment services. During 2017–2018, a total of 2,780 NSP clients with positive test results for HCV antibody were identified in the treatment database by their NIN. Of 494 who completed treatment and were tested for HCV RNA ≥12 weeks after completing treatment, 482 (97.6%) were cured of HCV infection. Following the launch of the elimination program, Georgia has made much progress in hepatitis C screening among persons who inject drugs; recent data demonstrate high cure rates achieved in this population. Testing at NSPs is an effective strategy for identifying persons with HCV infection. Tracking clients referred from NSPs through treatment completion allows for monitoring the effectiveness of linkage to care and treatment outcomes in this population at high risk, a key to achieving hepatitis C elimination in Georgia. The program in Georgia might serve as a model for other countries.

The Georgian Harm Reduction Network began operating and receiving hepatitis C testing data from NSPs in 2006. As of 2016, 16 NSPs were operating in 13 cities across Georgia. During 2017–2018, with additional resources provided by the Global Fund to Fight AIDS, Tuberculosis and Malaria, two additional NSP centers and eight mobile NSP units became operational, increasing coverage to approximately 50 of 79 municipalities countrywide. The Georgian Harm Reduction Network also provides diverse services* to persons who inject drugs to improve their health outcomes (*5*).

Persons who inject drugs and who test positive with a rapid HCV antibody test at NSPs are offered case management support and referred to authorized treatment sites for testing to confirm active HCV infection.^†^ Since 2017, those persons who agree to treatment referral are asked to provide their 11-digit NIN to the NSP so that further clinical management can be confirmed and documented in the national program treatment database. Once at the treatment center, those patients with confirmed infection are enrolled in the treatment program and, if eligible for treatment, prescribed a direct-acting antiviral regimen according to national treatment guidelines (*6*). Within 12–24 weeks of completing treatment, patients are instructed to return to the treatment site for HCV RNA testing to determine whether sustained viral response (i.e., virologic cure) was achieved. Demographics, diagnostics, and treatment outcomes are recorded in real-time in the national program treatment database.

For this analysis, program records from the Georgian Harm Reduction Network were reviewed to ascertain annual HCV antibody screening and positivity frequencies at NSPs during January 2006–December 2018 among persons who inject drugs; age group and sex distribution data were available from NSPs for 2015–2018. NSPs entered testing and service provision data into a database, which were validated by data management specialists at the Georgian Harm Reduction Network. Deduplication of test results was not conducted during 2006–2013 because of insufficient resources; during 2014–2018, deduplication of results was performed for each calendar year. Data for HCV antibody-positive persons who inject drugs who provided their NIN to NSPs during January 1, 2017–December 31, 2018, were linked to the national program treatment database to ascertain the hepatitis C care cascade, which summarizes the sequential steps in care. Because this analysis constituted a program evaluation, institutional review board oversight was not indicated.

During 2006–2018, NSPs provided 118,943 HCV antibody tests to persons who inject drugs, 48,228 (40.5%) of which were positive (Figure 1). During the years preceding program implementation (2006–2014), 32,738 (average 3,638 per year) tests were conducted; nearly half (49.6%; 16,247) were positive. Following implementation of the elimination program (2015–2018), the average number of antibody tests performed each year among persons who inject drugs increased approximately 500%, to 21,551. Among the 86,205 HCV antibody tests provided during this period, 31,981 (37.1%) were positive. Males accounted for 96.1% of tests, and persons aged 30–39 years were the most frequently tested age group (33.7%). In 2018, the HCV antibody prevalence among persons aged 18–29 years was 5.5%, the lowest among all age groups during 2015–2018. HCV antibody positivity was 37.8% among males and 24.0% among females tested at NSPs during 2015–2018.

**FIGURE 1:**
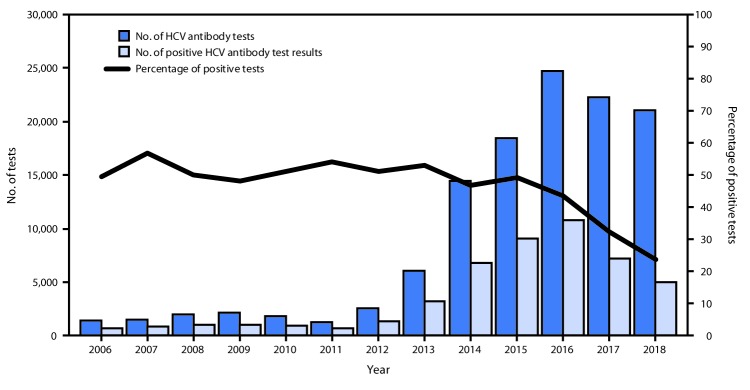
Number of tests for hepatitis C virus (HCV) antibody conducted and positive test results among persons who inject drugs — Georgian Harm Reduction Network, Georgia, 2006–2018

During 2017–2018, among 12,163 HCV antibody-positive test results from 11,424 clients at NSPs, 2,780 (24.3%) persons were identified by their NIN in the national treatment database, 1,626 (58.5%) of whom received a follow-up diagnostic test for active HCV infection (Figure 2). Among those tested, 1,370 (84.3%) had active HCV infection. Of those with active infection, 1,029 (75.1%) initiated treatment, 892 (86.7%) of whom completed treatment and were eligible for sustained viral response testing. Of these, 494 (55.4%) returned for sustained viral response testing, 482 (97.6%) of whom achieved cure.

**FIGURE 2:**
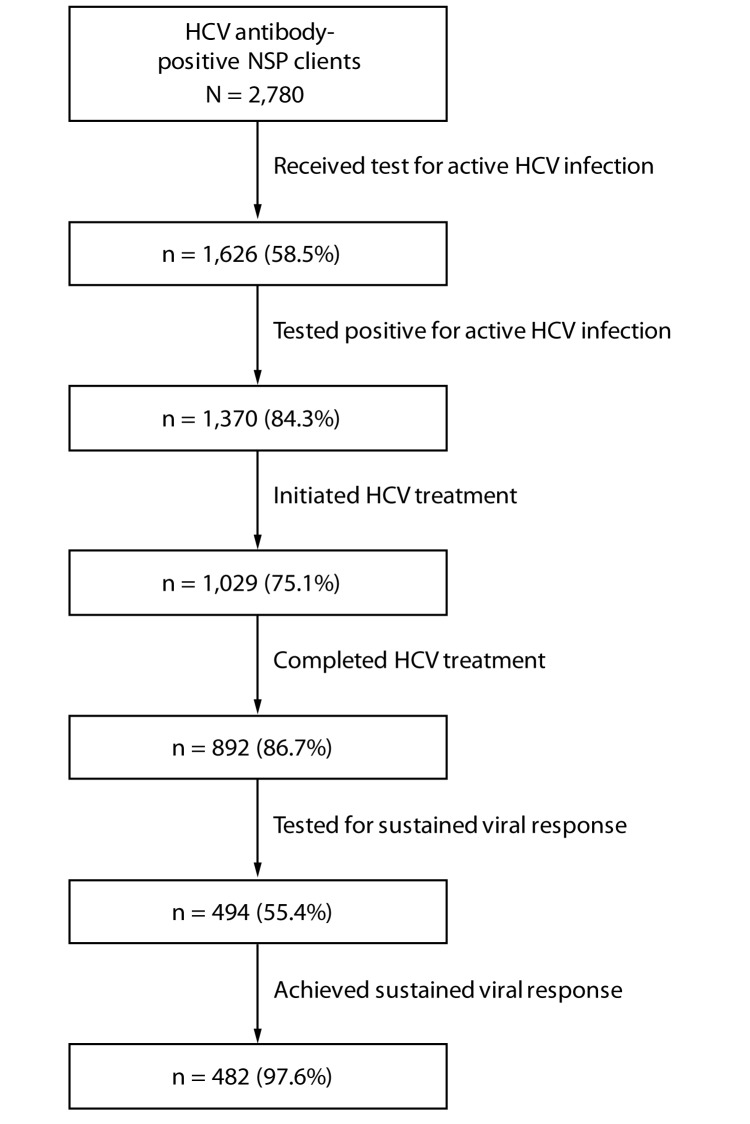
Hepatitis C virus (HCV) testing* and treatment outcomes among persons who inject drugs referred by needle and syringe programs (NSPs) to the national hepatitis C treatment program, as identified by their national identification numbers — Georgia, 2017–2018 * HCV RNA or HCV core antigen.

## Discussion

Hepatitis C testing at NSPs in Georgia is an effective strategy for identifying persons with HCV infection. During the 3 years following the launch of the elimination program in Georgia in 2015, the number of HCV antibody tests performed at NSPs increased nearly fivefold, and the number of persons with positive test results doubled, compared with the number with positive test results during 2006–2014. Further, voluntary use of the NIN among persons who inject drugs and receive services at NSPs permitted monitoring the linkage to care and treatment, as well as treatment outcomes, among this population at high risk. The number of tests performed annually at NSPs peaked in 2016, and the percentage of positive test results has been trending down since the launch of the elimination program in 2015. The reasons for the decrease in testing after 2016 are unclear but might represent a decreasing pool of persons who inject drugs and remain unaware of their HCV infection status. The decrease in the proportion of positive test results at NSPs during 2016–2018 suggests that a higher proportion of persons who inject drugs screened in recent years have not yet had exposure to HCV. This interpretation is supported by the finding that among all age groups, those aged 18–29 years had the lowest HCV antibody positivity prevalence in 2018 and might attest to the effectiveness of the prevention measures provided by NSPs. Given the estimate of approximately 50,000 persons who inject drugs in Georgia and that nearly 120,000 HCV antibody tests have been conducted at NSPs (with approximately 50,000 positive HCV antibody test results) since 2006, it is likely that the majority of persons who inject drugs in Georgia have been tested at least once for HCV antibody.

Fewer than one fourth of persons who inject drugs agreed to provide their NIN to NSPs for the purpose of tracking clinical outcomes. Stigma related to drug use and fear of adverse legal, social, and economic consequences might discourage persons from disclosing their NIN to NSPs before accessing hepatitis C care and treatment (*6*). To avoid revealing their injection drug use status in the national registry and treatment database, persons could opt to visit treatment sites without divulging their affiliation with NSP services. At present, no incentives are offered by NSPs to motivate persons to provide their NIN. Without the NIN, persons who inject drugs cannot be tracked throughout the cascade of hepatitis C care, and the degree to which elimination efforts are proceeding in this population is hard to ascertain. A study is currently underway to examine the feasibility and effectiveness of providing screening, care, and treatment services at NSPs.

The findings in this report are subject to at least three limitations. First, data from NSP screening and the treatment programs could not be independently verified and could be subject to data entry errors. Second, resources were unavailable to deduplicate NSP test records before 2014; thus, it is not known whether each HCV antibody test during 2006–2013 represented a single person screened. Finally, because only a small proportion of screening data from NSPs were linked to treatment data, this analysis could not fully assess the effectiveness of linkage from NSP screening to the national care and treatment program.

Strategies to engage persons who inject drugs in hepatitis C prevention, care, and treatment are needed to ensure elimination in Georgia. Lessons from Georgia could inform other countries with a high prevalence and similar epidemiology of hepatitis C.

**Summary**

**What is already known about this topic?**

Georgia, with a high prevalence of hepatitis C virus (HCV) infection and a high prevalence of injection drug use, launched a hepatitis C elimination program in 2015. Since 2006, needle and syringe programs (NSPs) have offered HCV antibody testing for persons who inject drugs.

**What is added by this report?**

Following the launch of the hepatitis C elimination program, the number of HCV antibody tests performed at NSPs has increased fivefold, and the number of persons with positive test results has doubled.

**What are the implications for public health practice?**

Hepatitis C testing at NSPs is an effective strategy for identifying persons with HCV infection. The program in Georgia might serve as a model for other countries.

## Footnotes

* Services provided though the Georgian Harm Reduction Network include distribution of sterile injecting equipment, condoms, and naloxone; voluntary counselling and testing for hepatitis C, human immunodeficiency virus, hepatitis B, and syphilis; peer-to-peer education; raising prevention awareness among persons who inject drugs; and advocacy for increased access to NSPs.

† Positive for HCV RNA or HCV core antigen.

## References

[1] Mitruka K, Tsertsvadze T, Butsashvili M, Launch of a nationwide hepatitis C elimination program—Georgia, April 2015. MMWR Morb Mortal Wkly Rep 2015;64:753–7. 10.15585/mmwr.mm6428a2 26203628 PMC4584859

[2] Hagan LM, Kasradze A, Salyer SJ, Hepatitis C prevalence and risk factors in Georgia, 2015: setting a baseline for elimination. BMC Public Health 2019;19(Suppl 3):480. 10.1186/s12889-019-6784-3 32326913 PMC6696670

[3] Stvilia K, Tsertsvadze T, Sharvadze L, Prevalence of hepatitis C, HIV, and risk behaviors for blood-borne infections: a population-based survey of the adult population of T’bilisi, Republic of Georgia. J Urban Health 2006;83:289–98. 10.1007/s11524-006-9032-y 16736377 PMC2527157

[4] Bemoni Public Union; Curatio International Foundation. Population size estimation of people who inject drugs in Georgia 2016. Tblisi, Georgia: Curatio International Foundation; 2017. http://curatiofoundation.org/wp-content/uploads/2018/02/PWID-PSE-Report-2017-ENG.pdf

[5] European Monitoring Centre for Drugs and Drug Addiction. Country overview, Georgia. Lisbon, Portugal: European Monitoring Centre for Drugs and Drug Addiction; 2015. http://www.emcdda.europa.eu/publications/country-overviews/ge

[6] Georgia Ministry of Health, Labour and Social Affairs. National hepatitis C virus elimination progress report Georgia, 2015–2017. Tblisi, Georgia: Georgia Ministry of Health, Labour and Social Affairs; 2019. https://www.moh.gov.ge/uploads/files/2019/Failebi/25.04.2019-1.pdf

